# FunCoup 6: advancing functional association networks across species with directed links and improved user experience

**DOI:** 10.1093/nar/gkae1021

**Published:** 2024-11-12

**Authors:** Davide Buzzao, Emma Persson, Dimitri Guala, Erik L L Sonnhammer

**Affiliations:** Department of Biochemistry and Biophysics, Stockholm University, Science for Life Laboratory, Box 1031, 171 21Solna, Sweden; Department of Biochemistry and Biophysics, Stockholm University, Science for Life Laboratory, Box 1031, 171 21Solna, Sweden; Department of Biochemistry and Biophysics, Stockholm University, Science for Life Laboratory, Box 1031, 171 21Solna, Sweden; Department of Biochemistry and Biophysics, Stockholm University, Science for Life Laboratory, Box 1031, 171 21Solna, Sweden

## Abstract

FunCoup 6 (https://funcoup.org) represents a significant advancement in global functional association networks, aiming to provide researchers with a comprehensive view of the functional coupling interactome. This update introduces novel methodologies and integrated tools for improved network inference and analysis. Major new developments in FunCoup 6 include vastly expanding the coverage of gene regulatory links, a new framework for bin-free Bayesian training and a new website. FunCoup 6 integrates a new tool for disease and drug target module identification using the TOPAS algorithm. To expand the utility of the resource for biomedical research, it incorporates pathway enrichment analysis using the ANUBIX and EASE algorithms. The unique comparative interactomics analysis in FunCoup provides insights of network conservation, now allowing users to align orthologs only or query each species network independently. Bin-free training was applied to 23 primary species, and in addition, networks were generated for all remaining 618 species in InParanoiDB 9. Accompanying these advancements, FunCoup 6 features a new redesigned website, together with updated API functionalities, and represents a pivotal step forward in functional genomics research, offering unique capabilities for exploring the complex landscape of protein interactions.

## Graphical Abstract

**Graphical Abstract:**
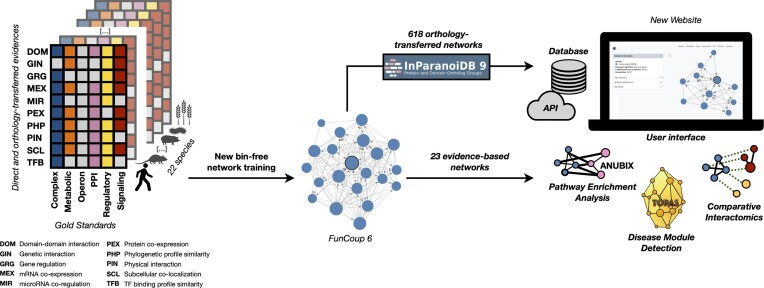

## Introduction

Elucidating protein interactions is fundamental for unraveling complex cellular processes. Current methodologies, ranging from precise but costly low-coverage experiments to cost-efficient yet error-prone high-throughput techniques like yeast two-hybrid, yield diverse interaction types, from direct physical to regulatory interactions. Existing databases like BioGRID (1), IntAct (2), ENCODE (3,4), SIGNOR (5), Reactome (6), PhosphoSitePlus (7), OmniPath (8) and NeDRex (9) store primary interaction data but may lack the comprehensive integration needed for a holistic understanding of the interactome. A robust framework for data integration is crucial for minimizing errors and capturing a comprehensive view of cellular processes and connecting them to functions and phenotypes.

Several frameworks, including FunCoup (10), STRING (11), HumanNet (12), PrePPI (13), GeneMania (14), HumanBase (15), IMP (16) and I2D (17) employ different machine learning techniques to integrate multi-omics data and represent interactomes with genes or proteins as nodes linked by functional associations. Researchers leverage the versatile capabilities of functional association networks across diverse domains, including hypothesis generation for both small-scale and large-scale data analyses, and contribute to the advancement of third party resources. The FunCoup database is built with a unique redundancy-weighted naïve Bayesian approach, combining 10 diverse data types and incorporating orthology transfer, to infer functional associations for 23 species. FunCoup has demonstrated superior performance in identifying disease genes using ORPHANET (18) gene sets (10) and ranked among the top three best performers in a benchmark of 46 networks (19). It facilitates hypothesis generation by revealing intricate gene relationships, as seen, e.g. in studies exploring the roles of genes post-Malat1 knockdown (20), uncovering co-expression patterns in disease contexts like CHARGE syndrome (21) and identifying relevant clusters in non-ossifying fibroma (22). FunCoup was integrated into a deep learning framework for predicting drug-target interactions (23), and in the development of the DiffBrainNet resource (24). FunCoup has further facilitated pathway enrichment analysis (EA) through tools like PathBIX (25), e.g. to investigate TP53 signaling disruptions in osteosarcoma (26) and the exploration of amyotrophic lateral sclerosis-related gene networks (27).

As the underlying data evolves, functional association networks require continuous framework updates. Previous versions of FunCoup, as well as other functional association networks, suffer from arduous training methodologies and coarse resolution due to binning techniques, resulting in low accuracy in link prediction and poor reproducibility. Furthermore, existing functional association databases generally lack gene regulatory interactions and are restricted in terms of species representation, creating gaps in the understanding of complex biological processes. Many resources have user interface limitations that impede the effective integration and use of advanced analytical tools. FunCoup 6 addresses these limitations by incorporating new methodologies and tools to enhance network inference and analysis. Key advancements include significantly expanded gene regulatory link coverage, a novel bin-free naïve Bayesian training framework, and a redesigned website. For 13 species, the number of regulatory links has increased substantially, with the human network alone featuring around half a million directed links, deepening our understanding of transcriptional regulation.

In addition to the MaxLink tool (28) for candidate gene prioritization, FunCoup 6 now includes the TOPAS algorithm (29) for identifying disease and drug target network modules. The resource is further enhanced with integrated KEGG (30) pathway EA utilizing the ANUBIX (31) and EASE (32) algorithms, enhancing its application in biomedical research. The unique comparative interactomics feature in FunCoup, which provides insights into network conservation across species, has been enhanced with a new search mode. This mode performs a network expansion in the other species with orthologs to the query, to identify interactors independently in each species. The new bin-free training was applied to 23 primary species, and networks for an additional 618 species in InParanoiDB 9 (33) were generated using a new orthology-based method. These improvements are complemented by a redesigned website and extended API functionalities, enhancing user accessibility and experience.

## Materials and methods

### Proteomes

In FunCoup 6, all proteomes from FunCoup 5 were updated using UniProt reference proteomes 2022-02 (34). We added the new species *Mycobacterium tuberculosis*. Identifier mappings necessary for integrating different types were extracted from UniProt ID mapping files.

### Gold standards

In FunCoup, gold standards serve as proxies for true interactions to assign confidence scores to protein associations, regardless of the evidence type. For this update, we used six gold standards, building on five from previous versions and adding a regulatory category to capture the interactions between transcription factors and target genes. The other gold standards include metabolic and signaling pathways, protein–protein interactions (PPIs), protein complexes and shared operons. When multiple sources contribute to a gold standard, their union is used. See Supplementary Figure S1 for a comparison of the number of links per gold standard between FunCoup 5 and FunCoup 6.

#### Complex

Protein members of the same complex, according to iRefIndex (v2022-08) (35), Corum (v2022-11) (36) and ComplexPortal (v2023-06) (37), were considered functionally related and were included in the complex gold standard. Some complexes included thousands of proteins, likely due to false associations. To minimize the impact of such noise during network training, we limited the size of complexes in the gold standard to a maximum of 100 proteins, following the largest complex size found in Corum.

#### Metabolic and Signaling

Metabolic and signaling links were extracted as fully connected networks between proteins belonging to KEGG (v101.0) metabolic and signaling pathways (38).

#### Operon

Shared operon links were extracted from OperonDB (v2022-02) (39).

#### PPI

PPI were extracted from iRefIndex (v2022-08). PPI must be supported by at least two experiments or be present in another gold standard. Similarly to the complex gold standard, we restricted the focus to physical interactions (PIN) with no more than 100 interactions per experiment.

#### Regulatory

We introduced the Regulatory gold standard TRRUST (v2) (40) RegNetwork (v2019-04) (41), RegulonDB (v2022-12) (42) and Yeastract (v2022) (43) to capture transcription factor regulatory interactions for (41). We used the union of TRRUSTv2 and RegNetwork for human and mouse links.

### Evidences

FunCoup 6 has 10 independent evidences, to capture functional association at multiple omics levels. Data are collected from public online databases and resources. For each data type, a scoring metric is used to measure the strength of association. Raw scores are subjected to transformation and normalization before entering the database.

#### DOM

DOMain–domain interaction (DOM) is based on pre-computed scores from UniDomInt (v1.0) (44). The UniDomInt score indicates support across source databases. Domain interactions were mapped to protein pairs using Pfam (v35.0) (45). DOM is computed as the ratio of the number of scored domain pairs *m* to the total possible pairs *N*, multiplied by the average of scores, weighted by the log of the sum of the degrees of the two interacting domains (Equation 1). *N* is calculated as the product of the number of Pfam domains in *A* and *B*.


(1) \begin{eqnarray*}DOM\left( {A,B} \right) &=& \frac{m}{N} \times \frac{{ \sum _{i = 1}^m \frac{1}{{log\left( {{{d}_a} + {{d}_b}} \right)}}UniDomInt\left( {a,b} \right)}}{m}\nonumber \\ \end{eqnarray*}

#### GIN

Genetic INteraction (GIN) was measured as a Spearman correlation of GIN profiles as reported in BioGRID (v4.4.219), with minimum five valid pairs. This cutoff was chosen to avoid spurious correlations often caused by low-coverage data, which can lead to multimodal, discrete distributions. A GIN profile represents how a gene interacts with many other genes. For example, if Gene A is mutated and tested with Genes B, C, and D, the results (e.g. how much the double mutations affect growth) form Gene A’s profile. If Gene B has a similar profile (similar effects with Genes C, D, etc.), it suggests that Genes A and B might be functionally related.

#### GRG

Gene ReGulation (GRG) was extracted from pre-computed Browser Extensible Data (BED) narrowPeak files in ENCODE (v131.0) for human, mouse, worm and fruit fly. We used the enrichment score of Irreproducibility Discovery Rate thresholded peaks that have undergone standardised ENCODE (human, mouse) or Robert-Waterston (mouse, fly) processing pipeline (46). To accommodate for technical variability, each dataset was normalized and then intersected with others. Redundant interactions were sorted by maximization of enrichment score. Pybedtools (v0.9.1) was used to handle gene annotations of detected peaks. Genomes GRCh38 and mm10 were downloaded from ENCODE, ce11 and dm6 were downloaded from Ensembl.

#### MEX

mRNA co-EXpression (MEX) was computed as a Spearman correlation of expression profiles for healthy samples (with minimum three valid pairs). Data were extracted from GEO and EBI Expression Atlas. Only pairs with absolute correlation larger than 0.5 were retained, and then min-max scaled between 0 and 1. MEX was not computed for homolog pairs. In FunCoup 6, we used DIAMOND (47) to identify homologous pairs and considered homologs those with default E-value below 0.001. We made a comparison of the number of homologs found with DIAMOND at bit score above 100 (i.e. which is similar to Blast (48) bit score) and E-value below 0.001 (Supplementary Figure S2).

#### MIR

microRNA co-Regulation (MIR) was computed as the Jaccard Index (JI) of microRNA regulating each protein pair, retaining only positive values. Here, we used previous data (49).

#### PEX

Protein co-EXpression (PEX) was measured on PaxDB (v5.0) quantitative mass spectrometry (QMS) data in two ways, by measuring the correlation profile over tissues (with minimum five valid pairs), and by measuring the JI of 25% top tissues each protein pair is expressed in, retaining only positive values, as it was previously done in FunCoup with the evidence QMS.

#### PHP

PHylogenetic Profile similarity (PHP) was assessed using phylogenetic profile data from all 640 species of InParanoiDB 9. An orthophylogram and a distance matrix were constructed using orthologs from the InParanoid species, where the distance between two species was computed as one minus the average fraction of orthologous proteins in both directions, i.e. 1 − [(number of orthologous proteins in species A / total number of proteins in species A) + (number of orthologous proteins in species B / total number of proteins in species B)] / 2. Subsequently, a profile was generated for each protein in the FunCoup species, with the presence of an ortholog represented by 1 and the absence by 0. To determine PHP scores for a specific species, the distance matrix was employed to construct a neighbour-joining tree rooted at the species of interest. For each species, and for every pair of proteins, we used the phylogenetic profiles to calculate the PHP score. This score was derived from the logarithm of the ratio between a ‘positive score’ and a ‘negative score’. The positive score represents the total branch length of the subtree encompassing all species that have orthologs for both proteins, divided by the total branch length of the entire tree. Conversely, the negative score represents the total branch length of the subtree containing species where only one of the proteins has an ortholog, divided by the total branch length of the entire tree. We use branch length to weight the conservation of gene pairs through evolution, with the idea that deeper, more ancient conservation represents a stronger signal of functional association than more recent conservation.

#### PIN

PIN was computed as the weighted average of the number of publications *n* supporting the interaction, as listed in IrefIndex (v2022-08) (35), with weights based on the logarithm of the interactions reported in each publication *PMID_i_*, downweighted by the logarithm of the total number of connections of proteins *A* and *B* (Equation 2). The degree *d_A_* represents the total number of interactions that protein *A* has in IrefIndex (*d_B_* for protein *B*), and these are used to increase the specificity of the detected interactions by accounting for proteins that have a high number of interactions.


(2) \begin{equation*}PI{{N}_{A,B}} = \frac{{ \sum _{i = 1}^n 1/log\left( {1 + \left| {PMI{{D}_i}} \right|} \right)}}{n} \times \frac{1}{{log\left( {{{d}_{A\ }} + {{d}_B}} \right)}}\end{equation*}

#### SCL

SubCellular co-Localization (SCL) was measured as semantic similarity of gene ontology (GO) keywords (v2023-03) (50). To compute similarity scores, we used the Wang et al. graph-based method (51), which uses the topology of the GO Directed Acyclic Graph structures.

#### TFB

Shared transcription factor binding (TFB) profile similarity was measured as the JI of transcription factors binding to each protein pair, retaining only positive values. Data were downloaded from TFLink (v1.0) (52).

### Orthology

Evidence types GIN, GRG, MEX, MIR, PEX, PIN and TFB are eligible for evidence transfer using orthologs, meaning that the species specific evidence of those types are transferred using orthologs from InParanoiDB 9 (33) to each of the FunCoup species, and is used as an additional source of evidence. Scores for many-to-one relationships (e.g. A1, B1 and C1 in species 1 are all orthologs of A2 in species 2 and pair with D1, an ortholog of D2) were averaged into a single score to avoid duplicates. Evidence types DOM, PHP and SCL are not transferred between species because they already contain cross-species information.

In FunCoup 6, we introduced the possibility to query networks for all 640 species available in InParanoiDB 9. This is done by transferring entire networks for each InParanoid species from their closest species among the 22 FunCoup networks using InParanoiDB 9 orthologs. The closest FunCoup species for each of the InParanoid species was obtained by taking NCBI taxonomy lineage information, and finding the FunCoup species closest to it in the taxonomy tree. For species where taxonomy information was missing from NCBI, or where ties occurred, i.e. a species was equally close to multiple FunCoup species, the species with the shortest distance in the orthophylogram for InParanoiDB9 was used. We used the unweighted pair group method to build a tree from distances calculated as 1 minus the fraction of orthologous proteins averaged over both directions for all InParanoiDB 9 species pairs. When transferring a network, each link in the original network was transferred to all possible pairs of orthologs in the target species, keeping the original link score. All transferred networks are available for download from the FunCoup website, and the transferred species can be queried in the FunCoup website using UniProt IDs. All query types and options are available when querying for a transferred species, but as KEGG pathway information is not available for all transferred species, pathway enrichment was deactivated. The orthology-based inference approach was evaluated by comparing the networks of the species *Bacillus subtilis, Escherichia coli, Methanocaldococcus jannaschii, Saccharomyces cerevisiae, Schizosaccharomyces pombe* and *Sulfolobus solfataricus*, with the evidence-based approach. For each of the six target species, an independent network was trained by deactivating orthology transfer from the target species evidence, and then an orthology-transferred network was inferred from that. The network comparisons were made in terms of JI and overlap coefficient (OC) similarity of nodes and links between the evidence-based and orthology-transferred networks.

### Bin-free naïve Bayesian network training

FunCoup 6 uses a bin-free redundancy-weighted naïve Bayesian framework to extract likelihoods of functional associations from high-throughput data and gold standards. For each species, raw scores were assigned to evidence types, including those transferred from orthologs of other species, and a log likelihood ratio (LLR) was computed. Likelihoods are obtained by modeling the probability density function of the raw scores using kernel density estimation (KDE) with Gaussian kernels. The Silverman's Rule (53) was used for bandwidth optimization. We used the gaussian_kde python function from the scipy.stats library (v1.10.0).

LLRs are computed by calculating the ratio of positive and negative likelihoods over a common range of raw scores (Equation 3),


(3) \begin{eqnarray*}LLR\left( x_{i} \right) = ln \frac {P(e_{(x_i)}| FC)} {P(e_{(x_i)}| \neg FC)}\end{eqnarray*}

where $x_i$ is one single data point in a continuous score range, and the likelihood distribution for each evidence $e$, given known functional associations $FC$ and unknown functional associations $\neg FC$, is computed as follows. Polynomial regression is used to model the LLR distribution within a constrained range, typically up to the 98th percentile, excluding outliers. Successful training requires a dataset with a range of 10^3^–10^6^ observations, and the polynomial regression must meet specific criteria, including an ${{R}^2}$ value exceeding 0.9 and a polynomial degree between 2 and 4. The resulting $\hat{LLR}$ function, expressed as a polynomial of the raw score $x$, is given by Equation 4, where ${{\beta }_0}$, ${{\beta }_1}$, ${{\beta }_2}$, ${{\beta }_3}$ and ${{\beta }_4}$ are the polynomial coefficients learned from the data. Throughout the text, we refer to the estimated $\hat{LLR}$ as LLR.


(4) \begin{eqnarray*}\hat{LLR}\left( x \right) = {{\beta }_0} + {{\beta }_1}x + {{\beta }_2}{{x}^2} + {{\beta }_3}{{x}^3} + {{\beta }_4}{{x}^4}\end{eqnarray*}

The LLRs for gene pairs with support by multiple datasets of MEX are weighted to avoid redundancy. The final $LL{{R}_{t\ }}$from evidence $E$ of type $t$ is the weighted sum of the LLRs from different datasets of ${{E}_t}$ (Equation 5).


(5) \begin{eqnarray*}LL{{R}_t}\left( {A,B} \right) = \sum \limits_k^{\left| {{{E}_t}} \right|} {{w}_{kt}}LL{{R}_{kt}}\left( {A,B} \right)\end{eqnarray*}

The different datasets ${{e}_{kt}} \in {{E}_t}$ are ranked by their $LL{{R}_{kt\ }}$in decreasing order. Then the Spearman's rank correlation ${{r}_{ki}}$ is used to measure the distance between each dataset ${{e}_{kt}}$ and ${{e}_{it}}$ as $\alpha [ {1 - max( {0,{{r}_{ki}}} )} ]$, with $\alpha$ being estimated from the noise in the data and set to 0.7 as optimized in previous releases. An additive sequential redundancy schema was implemented to reduce the amount of calculations, given the increase of data and resolution during the training since the previous release. For each gene pair $A,B$, LLRs are ranked in decreasing order and summed up, down-weighted by a decreasing factor ${{w}_{kt}}$, which starts at 1 and sequentially reduced to ${{w}_{kt}} = \ \alpha [ {1 - max( {0,{{r}_{ki}}} )} ]*\ {{w}_{it}}$ between each dataset ${{e}_{kt}}$ and ${{e}_{it}}$, with $i \,<\, k$.

Given the independence assumption of evidences, for each gene pair $A,B$ the final Bayesian score ($FBS$) is computed as a sum of the different LLRs of all evidence types ($LL{{R}_E}$), across all species, for each of the available gold standards (Equation 6).


(6) \begin{eqnarray*}FBS\left( {A,B} \right)\ = \ \sum \limits_t^{\left| E \right|} LL{{R}_t}\left( {A,B} \right)\end{eqnarray*}

### Link confidence PPV calculation

The link confidence estimation method involves assessing positive predictive value ($PPV$) across increasing $FBS$. In this method, the $PPV$ is computed as $TP/( {TP + FP} )$ for an equal amount of gold standard positive and negative examples, i.e. an $FBS$ of 0 when TP = FP corresponds to a $PPV$ of 0.5. Negative examples were sampled 30 times to capture variability, and the $PPV$ was computed over a range of 1000 threshold values using the precision_score function from the python sklearn.metrics library (v1.2.2). The $\hat{PPV}$ confidence for all other gene pairs is then estimated by fitting a logistic function to the $PPV$ versus $FBS$ relation (Equation 7), providing an empirical measure of the confidence of functional associations within each gold standard network. In this equation, $a$ represents the maximum value that the function approaches (typically 1), $b$ controls the steepness of the curve, and $c$ shifts the curve horizontally along the *x*-axis. Successful fitting requires the logistic regression to have an ${{R}^2}$ value exceeding 0.9. Throughout the text, we refer to the estimated $\hat{PPV}$ as PPV.


(7) \begin{eqnarray*}\hat{PPV}\left( x \right) = \frac{a}{{1\ - \ {{e}^{ - b\left( {x - c} \right)}}\ }}\end{eqnarray*}

For each species, the final FunCoup network corresponds to the union of the gold standard networks with maximum PPV. Only links with PPV ≥ 0.85 are kept. In the updated scoring system of FunCoup, we introduce the concept of gold standard-based PPV (gsPPV) to integrate gold standard links more effectively. gsPPV values were assigned based on the number of gold standards supporting a link, with the values ranging between the minimum and maximum PPV stored in the database. Specifically, gsPPV was set to 0.85 for links supported by one gold standard, 0.90 for two, 0.95 for three and 1 for more than three. This is because most species have support from up to four gold standards (Complex, Metabolic, PPI and Signaling). However, if a link has a PPV value, estimated based on evidence, that is exceeding the gsPPV value, the gsPPV value is discarded.

### Network benchmark

To evaluate the performance of FunCoup 6, we compared its human network against FunCoup 5, HumanNet v3, and STRING v12 using ORPHANET gene sets that contain at least 10 genes. To ensure compatibility with each network's gene vocabulary and convert gene and protein identifiers to Gene Names, we utilized the UniProtKB UP000005640 file, v2021-02 for FunCoup 5 and v2022-02 for FunCoup 6, and HumanNet v3. For STRING v12, we used 9606.protein.aliases.v12.0.txt. For each of the 110 resulting distinct gene sets, we split disease-associated genes into two halves, mapping them to networks and evaluating their recovery using Random Walk with Restart (RWR). The RWR algorithm was executed using the dnet::dRWR function from the R (version 4.3.2) package dnet, with starting probability of 0.75 from half of the genes in each geneset. Performance was assessed by computing the Area Under the Receiver Operating Characteristic (AUROC) curve. True positives were defined as the remaining half of the disease-associated genes for the investigated disease, while all remaining genes were classified as false positives. To avoid bias in the results, we created 30 distinct splits of each of the 110 gene sets. We quantified the Performance Gain $PG$ to evaluate how the real network compares to randomized networks (Equation 8),


(8) \begin{eqnarray*}PG\left( X \right)\ = \frac{{AURO{{C}_{{{S}_{30}}}}\left( X \right) - \ AURO{{C}_{R{{S}_{900}}}}\left( X \right)}}{{AURO{{C}_{R{{S}_{900}}}}\left( X \right)}}\end{eqnarray*}

where $X$ is one gene set in the ORPHANET library; $PG$ is the performance gain and $AUROC$ is the AUROC curve; $\ AURO{{C}_{{{S}_{30}}}}_{}( X )$ is the median AUROC computed for 30 splits of the geneset $X$; $AURO{{C}_{R{{S}_{900}}}}( X )$ is the median AUROC computed for 30 splits of the geneset $X$, under 30 independent randomizations of the network.

The benchmark was conducted under five distinct scenarios, by selecting the top 100, 250, 500, 750 thousands and 1 million of the highest confidence links for each network. Randomization of networks was achieved using the igraph::rewire() function with keeping_degseq() mode. This randomization process preserves the node degree distribution of the networks and was performed without allowing for self-interactions (loops = False) and with the number of edge swaps equal to the size of the network.

The website

### Implementation of FunCoup 6

FunCoup 6 was built in Python (v3.10.8) in a version-controlled conda (v23.9.0) environment, using the Django (v4.1.1) framework. All data are stored in a PostgreSQL (https://www.postgresql.org/) database. Bootstrap (https://getbootstrap.com/) frontend framework was used as a base for the frontend components, and D3.js (https://d3js.org/) was used as a base for drawing a network for each query input. The FunCoup 6 application is run and deployed in a Docker (https://www.docker.com/) container, managed with Docker Compose (version 2.17.2).

### Network search algorithms

In FunCoup 6, the search capabilities have been significantly expanded beyond the previous two methods: querying genes as a group and querying each gene independently. The default search mode is group search. When using this mode, the database is queried with a set of genes, and the top N genes most connected to the query set are added. Activating the option to prioritize common neighbors increases the likelihood of selecting genes that interact with multiple query genes. In the independent search mode, the database is queried separately for each gene, and at each expansion step, the top N most connected genes for each individual query gene are added. Today, users can also use Maxlink and TOPAS for network queries. Maxlink identifies candidate genes linked to query genes, retaining those with significant interactions according to a hypergeometric test. It was previously accessible through a separate search page that used the now-deprecated Maxlink website, and has been fully integrated into the FunCoup framework. This integration allows Maxlink to be used in conjunction with all other search filters, settings, and restrictions available in FunCoup. These include comparative interactomics and querying specific gold standard networks and evidence types. In addition to Maxlink, the TOPAS algorithm has been added as a new network querying option. TOPAS is designed for detecting modules within networks and has proven effective in identifying biologically relevant disease modules. It can be used for queries involving multiple genes and can be combined with other search settings, including comparative interactomics. The comparative interactomics feature has also been redesigned to support different search strategies. Users can now choose to align orthologs only, which identifies the orthologs of the query network. Alternatively, they can independently query each species network and subsequently draw orthology relationships. There is also an option to use species-specific evidence exclusively.

### Pathway enrichment analysis

Pathway EA is a tool for interpreting gene sets that we can use to identify key biological functions and processes impacted by a group of genes, such as those belonging to a FunCoup subnetwork. Two newly integrated algorithms, EASE and ANUBIX are available on our website to identify network crosstalk between subnetwork genes and KEGG pathways. We have integrated the EASE algorithm, which belongs to the OVerlap Analysis (OVA) class of EA methods. EASE tests the proportion of subnetwork genes in KEGG pathways against a discrete probability distribution model and extracts one-sided *P*-values. It uses a conservative modification of Fisher's exact test by subtracting 1 from the overlap count. ANUBIX is a Network Enrichment Analysis (NEA) method. NEA methods evaluate the interconnectivity between a query gene set and a pathway within a functional association network. This evaluation employs either a parametric or a permutation-based approach to determine the statistical significance of the observed interconnectivity. ANUBIX assesses the significance of crosstalk using a beta-binomial distribution, tailored to each pathway and query gene set through a degree-aware resampling process. The analysis with ANUBIX can be further explored by seamless redirection to PathBIX (25), a dedicated web-based application. For these analyses, we use pre-computed statistics on FunCoup networks with confidence link scores above 0.95.

## Results

### Database content

The primary focus of FunCoup is to identify functional associations between proteins, which represent interactions where proteins work together to perform a common biological function. These functional associations can take various forms, including PINs, regulatory interactions, or co-participation in complexes or pathways. FunCoup employs different evidences of seven broad types (co-expression, co-localization, co-evolution, co-regulation, DOM, GIN and PPI) to infer these associations (Table 1). To assign confidence scores to these interactions, FunCoup uses six gold standards that act as proxies for true interactions, covering metabolic and signaling pathways, PPIs, protein complexes, shared operons and transcription factor regulatory interactions. Each link is annotated with a confidence score, based on a LLR framework, that reflects the probability of the interaction being correct. LLRs are available for each evidence type and species, and links are enriched through transfer via orthologs from InParanoiDB. Contributions from both species and evidence types can be customized on the FunCoup website, allowing users to recompute confidence scores, for example by selecting only PIN from a single species as the evidence source. The novel aspects introduced in FunCoup 6 are summarized in Supplementary Table S1.

**Table 1.** Summary of FunCoup evidence types^a^

Type	Description	Source
DOM	Domain-domain interaction	UniDomInt
GIN	Genetic interaction	BioGRID
GRG	Gene regulation	ENCODE
MEX	mRNA co-expression	GEO, EBI
MIR	microRNA co-regulation	microRNA.org
PEX	Protein co-expression	PaxDB
PHP	Phylogenetic profile similarity	InParanoiDB
PIN	Physical interaction	IrefIndex
SCL	Subcellular co-localization	Gene Ontology
TFB	Shared TF binding	TFLink

^a^Details of the particular datasets used, with links to data sources are available on the Statistics page on the website.

### Bin-free network training and updated data

In order to derive functional association likelihoods from high-throughput data and gold standards, FunCoup 6 employs a new bin-free naïve Bayesian framework that uses KDE and polynomial regression to compute log-likelihood ratios, and a new weighted redundancy schema to extract a FBS for each link and gold standard (Supplementary Figure S3). This method requires the data size and regression quality to meet specific thresholds for reliable results. A total of 2 969 combinations of gold standards and evidences met these requirements, accounting for 34% of all possible combinations across all species (Supplementary Figure S4). The majority of unsuccessful training was due to lack of data (i.e. too few gold standard examples to extract likelihoods).

In FunCoup 6, we renamed the evidence QMS to PEX and updated the data sources for the evidences PEX, PHP, PIN and SCL (Supplementary Table S2). To achieve broader coverage and more representative expression data for healthy individuals, we utilized different databases for GIN, MEX, MIR and TFB, and integrated a new evidence GRG for regulatory interactions. Due to the new training framework requiring continuous data, we made several changes to the evidence scoring methods, which are summarized in Supplementary Table S2.

The new link confidence estimation method in FunCoup calculates PPV across varying FBS by using gold standard links as positive examples and an equal number of random pairs as negative examples, then fitting a logistic function to the PPV vs FBS relation to provide an empirical measure of the confidence of functional associations. This method requires data and regression to meet specific criteria for accuracy (Supplementary Figure S5). A total of 57 gold standards have met these requirements, accounting for 83% of all networks across all species (Supplementary Figure S6). The most common reason for failed PPV extraction was poor fitting (*i.e*. logistic regression fit with ${{R}^2} \,<\, 0.9$).

### Evidence-based networks for 23 species

In FunCoup 5, we provided functional association networks for 22 species, composed of 17 eukaryotes, 2 bacteria, 2 archaea and the virus SARS-CoV-2. In FunCoup 6, we added one more bacterial species, *Mycobacterium tuberculosis*, reaching a total of 23 species. SARS-CoV-2 was built as a host-virus interactome together with human proteins, and was updated to the latest UniProt reference proteome (34) and IrefIndex release of interactions (35), resulting in a total of 2 879 links, of which 2 833 are links between 17 reference proteins and 1 924 human proteins and 46 are links between viral proteins only.

**Figure 1.**
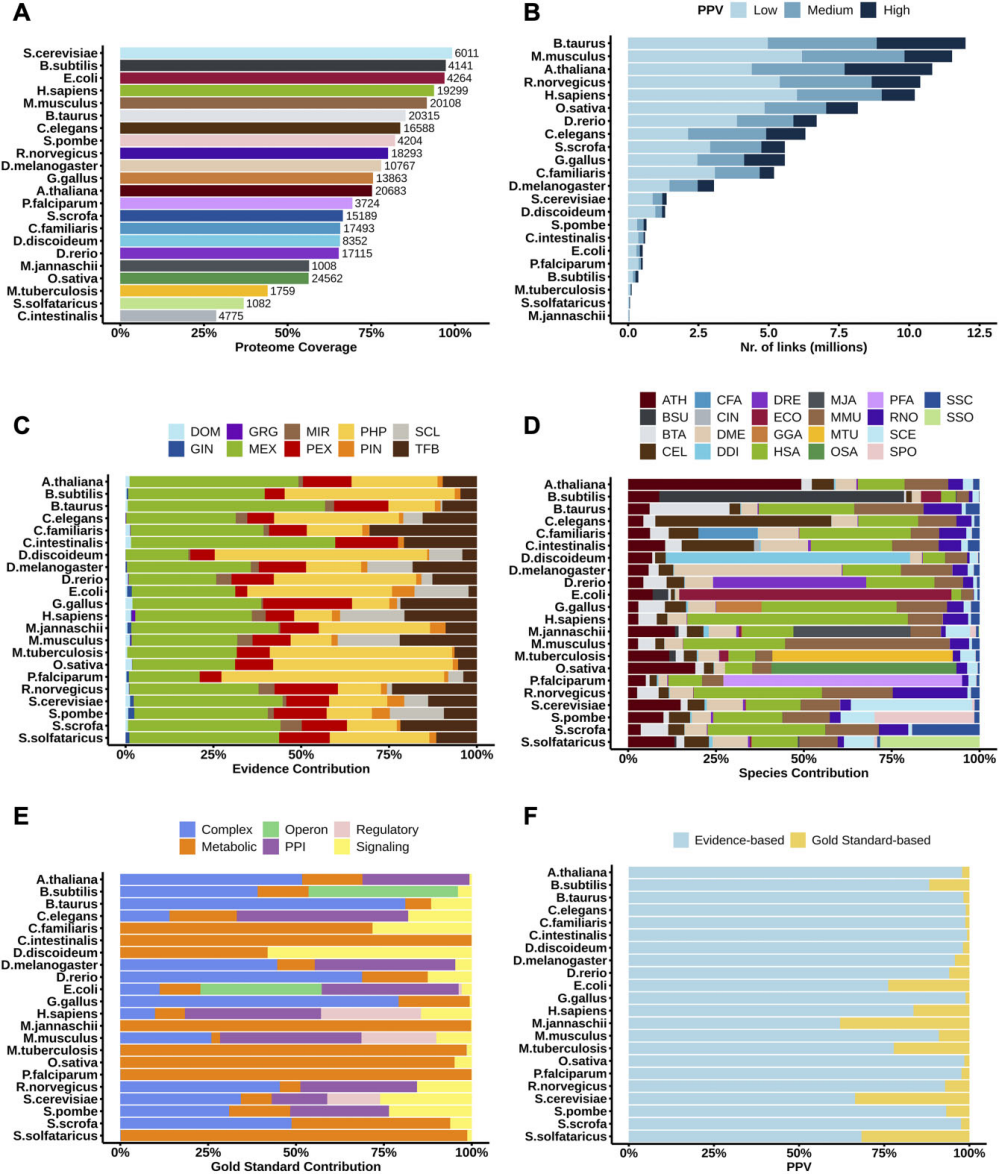
**FunCoup 6 statistics**. (A) The proteome coverage of the networks for each of the species in FunCoup 6. (**B**) The number of links for different PPV confidence cutoffs for each of the species in FunCoup 6. Low PPV confidence is PPV between 0.85 and 0.9, medium is PPV between 0.9 and 0.95, high is PPV above 0.95. (**C**) Contribution of evidence types to the networks of each FunCoup 6 species. The sum of LLRs is normalized so it sums to 1 per species. Evidence data types are: DOM: domain-domain interaction, GIN: genetic interaction; GRG: gene regulation; MEX: mRNA co-expression; MIR: microRNA co-regulation; PEX: protein co-expression; PHP: phylogenetic profile similarity; PIN: physical interaction; SCL: subcellular co-localization; TFB: shared transcription factor binding. (**D**) Evidence contribution from other species in each FunCoup 6 network. (**E**) Contribution of gold standard to the networks of each FunCoup 6 species. The number of links is normalized so it sums to 1 per species. (**F**) The percentage of links with gold standard- and evidence-based PPV.

The proteome coverage of the networks for each species can be seen in Figure 1A. The coverage has increased from FunCoup 5 for all species except *C. intestinalis* which decreased by thirteen percentage points. The proteome coverage exceeds 50% for all species except *S. solfataricus* and *M. tuberculosis* where it remains relatively low (37–44%), due to insufficient data for these less studied species.

The total number of functional associations has increased from 60 999 771 in FunCoup 5, to 101 054 256 in FunCoup 6. The distribution of links over different confidence score cutoffs for each species can be found in Figure 1B. For a majority of the species, the number of links in the network has increased in comparison to FunCoup 5, although for 3 species (*C. familiaris, C. intestinalis, and D. rerio*) we can see a decrease in the number of links.

The positive relative contribution of evidence types to the total FBS per species can be seen in Figure 1C. While the overall distribution of evidence types remains similar, MEX has decreased in its contribution compared to previous releases, partly due to more stringent criteria for correlation and homology, with PHP now taking a more prominent role due to its large coverage. PIN had limited coverage and a highly discretized score distribution in FunCoup 5, resulting in predominantly low or high scores, providing only a small contribution in all species except in *E. coli, S. cerevisiae and S. pombe*. In FunCoup 6, the PIN contribution remains small but is more uniformly distributed across species, with much smaller contributions in those three species. TFB has been notably boosted compared to the previous release. Similarly, GIN and DOM have also received enhancements, while MIR remains largely unchanged. However, these three evidences play a minimal contributing role overall.

Figure 1D illustrates the positive relative contribution of orthology transfer from other species to the total FBS of each network. Similar to previous FunCoup versions, the majority of species obtain more than 50% of their evidence through orthology transfer from other species.

For each species, the final FunCoup network corresponds to the union of the gold standard networks, keeping the maximum PPV. Only links with PPV ≥ 0.85 are kept. Figure 1E demonstrates that the contributions of various gold standards, such as Complex, Metabolic, PPI and Signaling, vary across different species. While some species rely heavily on one or two types of gold standards, others display a more balanced mix. Operon was only used for bacteria. A new Regulatory gold standard was added for *H. sapiens, M. musculus, E. coli* and *S. cerevisiae*.

Out of the over 100 million links, 7 383 393 are Gold Standard links. The updated FunCoup scoring system integrates gsPPV by assigning values based on the number of supporting gold standards, prioritizing manually curated evidence over lower evidence-based PPV values for higher prediction accuracy. Figure 1F shows the percentage of links with gold standard- and evidence-based PPV. The majority of links (65–99%) were assigned an evidence-based PPV.

### Directed links

**Figure 2.**
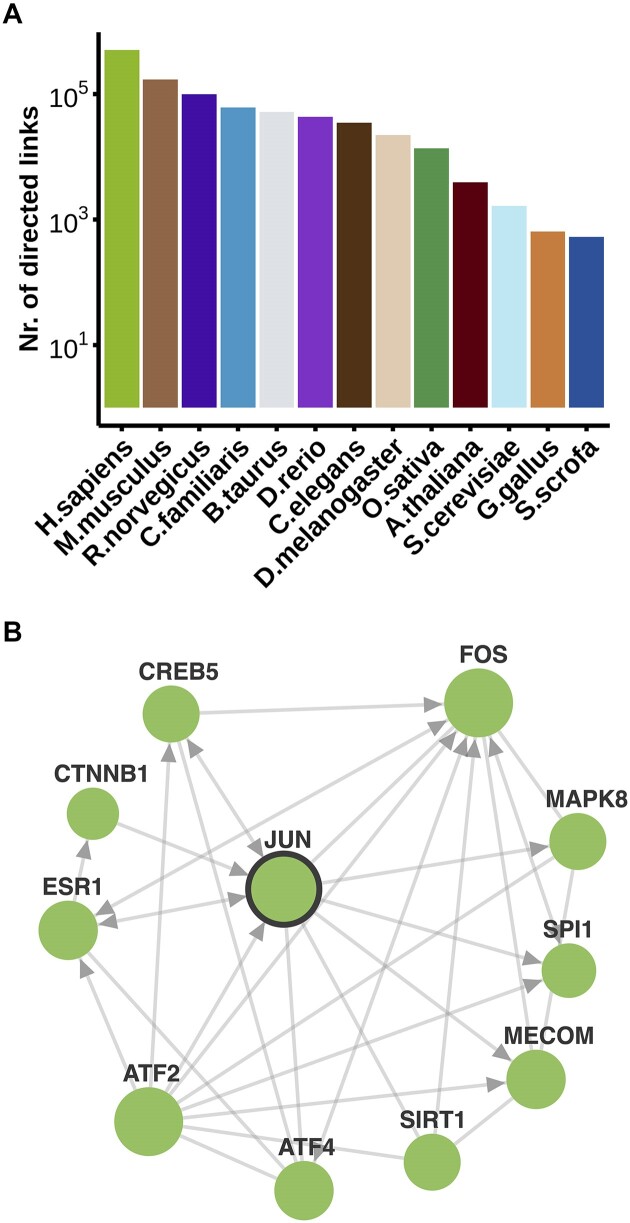
**Directed links in FunCoup 6 and a JUN regulatory subnetwork in human**. (A) The number of directed links in 13 FunCoup species. (**B**) A *JUN* FunCoup subnetwork, with 11 nodes and 30 links, of which 23 are directed to exhibit GRG. The database query (link) was performed on the website with the following parameters: query gene ‘JUN’; links and direction confidence threshold of 0.95 and 1; expansion depth 1; max 10 neighbors. Genes were searched as a group, with the prioritization of neighbors option activated. In the network illustration: the query gene is highlighted with a bold outline, the size of the nodes is proportional to the degree of connectivity, and nodes are colored in green using a new website feature.

In FunCoup 5, we introduced directed regulatory links at a very modest scale in human, inferred from ChIP-seq data, to denote transcription factor to target gene binding. In FunCoup 6, significant advancements were made in expanding regulatory networks across 13 species, for a sum of 964 037 directed links with positive GRG (Figure 2A). Positive GRG is sufficient to make the link directed, and it can be found for any gold standard. In the human network, there are 873 transcription factors interacting with 15 018 target genes, for a total of 501 720 directed links, 450 times more than in FunCoup 5. As an example, Figure 2B shows a subnetwork around the transcription factor JUN, known to play a significant role in cellular stress responses, apoptosis, and proliferation (54). Among its highest confidence interactions, links to MAPK8, ESR1, CREB5 and CTNNB1 were previously reported in TRRUST and/or RegNetwork. Other interactions, like those with ATF2, MECOM and SPI1, are highly supported by the GRG evidence, suggesting further complexity in the JUN regulatory network, for which support in the literature exists (55,56). Overall, the links in the network were inferred with Regulatory and PPI as the strongest gold standard and PIN as the strongest evidence type.

### Network search algorithms

There are four algorithms in FunCoup 6 to search the network to expand a set of query genes to neighboring genes: Independent, Group, MaxLink, and TOPAS. To exemplify these, we applied them to a query of *Biliary liver cirrhosis* genes, as described in a published collection of 70 disease gene sets (57). This gene set (21 genes) is highly disconnected in the human network (6 links, Supplementary Figure S7A) when we use high-confidence links in FunCoup (PPV ≥ 0.95), indicating that many relevant genes might be missing from the query.

In the independent search (Supplementary Figure S7B), each gene is queried separately, expanding the network by adding the top most confident interactors for each gene. This method generally results in a rapid increase in network size. The group search method (Supplementary Figure S7C), especially when the ‘common neighbor prioritization’ option is activated, tends to expand the network to a lesser degree. It adds top interactors with the highest number of interactions above the cutoff to the entire query group. This approach identified immediate neighbors that interact with multiple query genes, thus forming a cohesive subnetwork, but not as homogeneously throughout the genes.

The MaxLink algorithm (Supplementary Figure S7D) prioritizes genes based on the statistical significance of the connections to the query. However, it here struggled to identify new candidates because the input genes are largely disconnected and have few shared links to other genes. In contrast, the TOPAS algorithm (Supplementary Figure S7E) excelled in this context. TOPAS identified 11 connectors for 20 seed/query genes, 2 of which (i.e. B2M and PDGFR) were previously shown to be relevant in liver cirrhosis (29), demonstrating its utility in constructing biologically connected disease modules from a sparse and incomplete gene set.

### Comparative interactomics analysis

**Figure 3.**
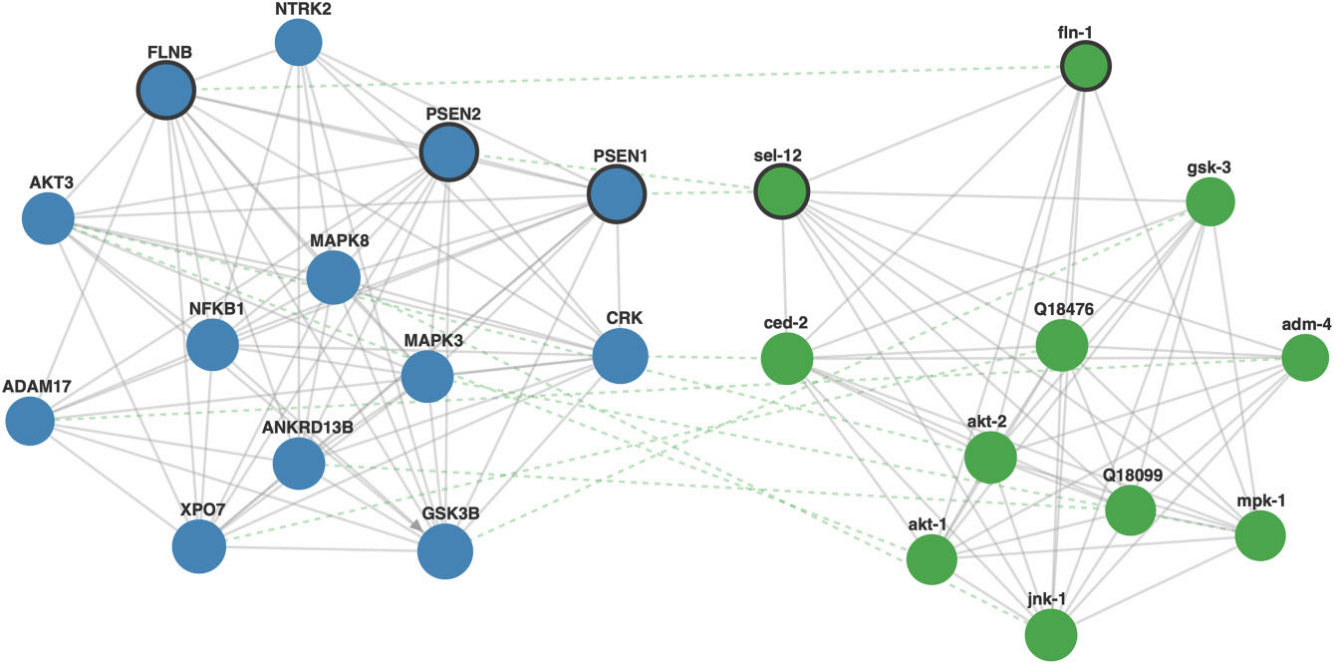
**Comparative interactomics analysis of an Alzheimer's disease related gene set**. Mixed subnetworks of the genes PSEN1, PSEN2 and FLNB in *H. sapiens* (blue, left) and *C. elegans* (green, right) showing functional (gray solid lines) and ortholog links (green dashed lines). The database query (link) was performed via the website with the following parameters: query genes ‘PSEN1,PSEN2,FLNB’; query species *H. sapiens* and comparative species *C. elegans*; links and direction confidence threshold of 0.5 and 1; expansion depth 1; max 10 neighbors. Genes were searched as a group, with the prioritization of neighbors option activated. In the network illustration: query genes are highlighted with bold outlines, and the size of the nodes is proportional to the degree of connectivity.

One of the unique functionalities of FunCoup is its comparative interactomics search to investigate network conservation between species. The redesigned comparative interactomics allows users to either align orthologs of the expanded query network only, or to search each species with orthologs to the query and expand separately in each species. By default, only species-specific evidence is used to avoid conservation from orthology-based evidence. In Figure 3 we illustrate comparative interactomics by searching in *C. elegans* for the network conservation of Presenilin-1 (PSEN1), Presenilin-2 (PSEN2), and Filamin B (FLNB), three genes implicated in Alzheimer's disease (AD) in human (58). PSEN1 and PSEN2 are involved in the processing of amyloid precursor protein and FLNB in cytoskeletal organization. PSEN1 and PSEN2 are orthologs to sel-12, and FLNB to fln-1. Of particular interest are ‘rectangular’ constellations, which consist of two functional associations and two ortholog links, here formed by PSEN1-CRK and sel-12-ced-2. CRK has not previously been associated with AD, but it is a signaling protein involved in cell adhesion that is associated to the physiological functions of amyloid precursor proteins and the formation of neurotoxic amyloid-ß peptide aggregates, which are considered to play a central role in the etiology of the disease (59–61). The ortholog in *C. elegans*, ced-2, is involved in cell death which is a hallmark of AD. Thus, investigating ced-2 in the worm offers advantages such as using its genetic tools for precise studies, gaining insights into the role of CRK in AD pathways involving PSEN1, and revealing evolutionarily conserved mechanisms relevant to disease research.

### Network benchmark

**Figure 4.**
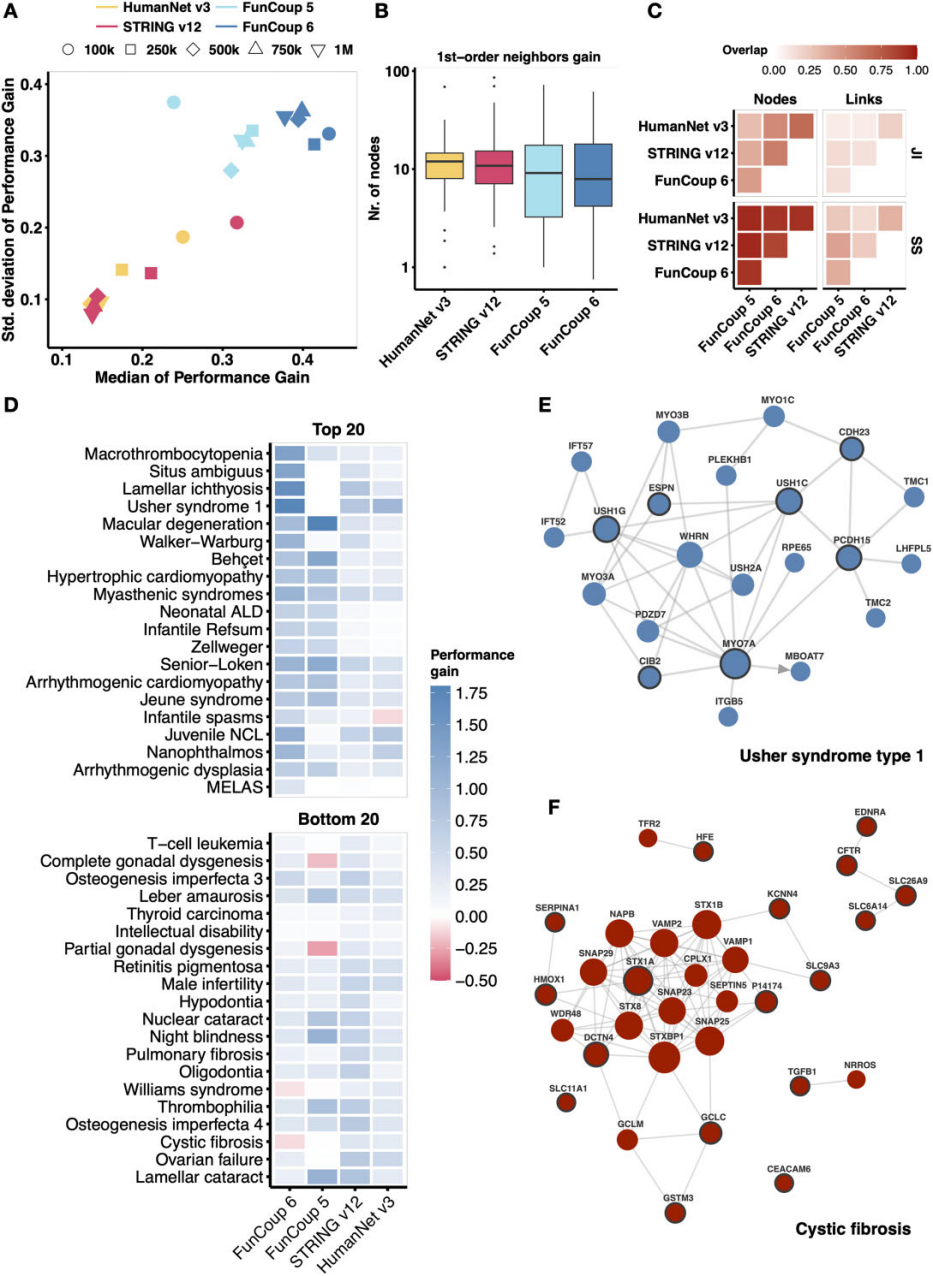
**Performance evaluation of disease gene sets from ORPHANET across varying network configurations**. (A) Performance gain is quantified as the difference in AUROC curve between real networks and their null counterparts. The assessment was done using a top fixed number of links: 100 000, 250 000, 500 000, 750 000 and 1 000 000, and summarized in a scatter plot with colors and shapes indicating networks and links cutoff. (**B**) The distributions of ORPHANET gene set sizes after mapping them onto the networks using top 100 000 most confident links, and first-order neighbor node gain, expressed as the ratio of non-query nodes that are first-order neighbors to the query genes, and the number of query genes. (**C**) Similarity between the networks expressed as JI and SS coefficient for nodes and links. (**D**) Heat map showing the performance gain values for the 20 top and bottom performing ORPHANET genesets in FunCoup vs HumanNet/STRING. These genesets were selected after sorting by Performance Gain fold-change, which is computed as the difference in performance gain in FunCoup 6 and the maximum performance gain with STRING or HumanNet. The gene set names were shortened for visualization purposes. (**E**) The FunCoup 6 subnetwork of Usher syndrome type 1 (i.e. USH1K, CDH23, USH1C, USH1G, MYO7A, PCDH15, USH1E, USH1H, CIB2 and ESPN) with overall highest performance gain in FunCoup 6. The subnetwork is composed of 22 genes and 41 links when querying the full network (PPV ≥ 0.85) with genes as a group, with expansion step of 1 and 15 nodes per expansion step. USH1E, USH1H and USH1K are not in the network. (**F**) The FunCoup 6 subnetwork of Cystic fibrosis (i.e. SERPINA1, SLC26A9, SLC6A14, SLC9A3, CEACAM3, CEACAM6, EDNRA, GSTM3, HMOX1, GCLC, HFE, CFTR, TGFB1, DCTN4, CLCA4, STX1A, KCNN4, SLC11A1 and MIF), composed of 34 genes and 89 links. The size of the nodes is proportional to the degree of connectivity. In the network illustrations: the query genes are highlighted with a bold outline, the size of a node is proportional to its degree of connectivity and the thickness of the links is proportional to the confidence.

To compare the performance of FunCoup 6 with FunCoup 5, HumanNet v3 and STRING v12, we followed an approach taken in previous large-scale benchmarks (19,62). We split disease-associated genes from 110 distinct ORPHANET gene sets into two halves and assessed their recovery using RWR, measuring Performance Gain (PG) across five scenarios with varying numbers of high-confidence links (Figure 4A). PG quantifies how the real network compares to randomized networks. FunCoup 6 shows superior performance compared to the other networks. Despite a larger standard deviation, FunCoup 6 exhibits higher median performance gain. The full distribution of PG at the various cutoffs is shown in Supplementary Figure S8.

To investigate the reasons behind the different performances in our benchmark, we analyzed network properties of the ORPHANET gene sets. We restricted the following analysis to the benchmark using only top 100 000 links, given that most networks have best average performances at this cutoff. FunCoup 5 has the worst performance, and that is because of a significantly lower genome coverage at high confidence links. Gene sets with low performance gain in an RWR test tend to be those that are fairly well connected to the rest of the network. This means that these gene sets experience a significant increase in nodes when adding first-order neighbors to the ORPHANET subnetwork. Specifically, if we measure the gain in terms of nodes when adding first neighbors to the query set, measured as ratios of non-query nodes and query nodes, we observe that FunCoup 6 has the lowest median gain of nodes at all cutoffs (Figure 4B and Supplementary Figure S9). This suggests smaller expansion and more specific connections, which benefits the RWR by preventing the flow from dispersing too widely across the network. See Supplementary Figure S10 for an analysis of other ORPHANET subnetwork properties.

We also measured how similar the networks are in terms of common nodes and links (Supplementary Figure S11). HumanNet v3 (1 125 484 links) and STRING v12 (6 857 369 links) are the most similar networks, with STRING v12 including about 60% (Szymkiewicz–Simpson (SS) coefficient of 0.58) of the links present in HumanNet v3. FunCoup 6 includes about half of the links in FunCoup 5 (SS = 0.48) and 40% of the links present in HumanNet v3. If we compare the networks using only top 100 000 links, overall FunCoup 6 has the most different connections, with a JI of 0.08, 0.13 and 0.14 with HumanNet v3, STRING v12 and FunCoup 5 (Figure 4C).

To highlight the fact that networks differ and are therefore complementary, with gene sets performing differently across them, we isolated the subnetworks with the highest and lowest average performance gain for FunCoup 6 versus the maximum performance gain for either STRING v12 or HumanNet v3. Figure 4D shows the performance gain values for these top and bottom 20 ORPHANET genesets, illustrating the variability in performance across different networks.

To visually distinguish between a well-performing subnetwork and a poorly performing one, we compared the in FunCoup 6 best-performing gene set (Usher syndrome type 1) with the worst-performing gene set (Cystic fibrosis) on average (Figure 4E and 4F). The difference in performance gain between the two gene sets can be attributed to network structure and the distribution of gene importance. In Usher syndrome type 1 the query genes are central and highly connected, facilitating effective network propagation. This structure allows the network to efficiently prioritize the relevant genes. Conversely, in Cystic fibrosis the genes are highly connected but primarily to other genes with even higher degrees. Additionally, some nodes in this gene set are quite disconnected from the overall network, further diminishing the effectiveness of network propagation and resulting in lower performance.

### Orthology-transferred networks for 618 species

In FunCoup 6, we enabled network queries for all species in InParanoiDB 9 by transferring the network from the closest of the primary FunCoup species using InParanoiDB 9 orthologs. To evaluate the orthology-transferred networks, we compared six species, grouped into three pairs of closest species: *B. subtilis – E. coli, S. cerevisiae – S. pombe and M. jannaschii – S. solfataricus*. The networks of these species were compared with those generated by the evidence-based approach (Supplementary Figure S12), revealing Jaccard Indices up to 0.7 and OCs up to 1.0 of nodes and links, indicating a close approximation despite the lower coverage of the transferred networks. An exception was observed for *S. pombe*, which had higher link coverage when transferred from *S. cerevisiae* orthologs, likely due to the large amount of data available for the latter.

### The website

The latest update to FunCoup 6 introduces several key improvements to enhance user experience and functionality. The website frontpage has been completely redesigned for a more intuitive user experience, featuring a condensed and more intuitive advanced search function. To address ambiguities in the gene names in the query set, we first perform a database search that identifies any genes that could not be found or have ambiguous names. An intermediate page then lists these genes, allowing users to select the intended genes. The network view utilizes the D3.js library, now fully integrating with the interaction table, so hovering over table rows highlights corresponding links in the network. Additionally, the side panel next to the network enables extensive customization options, allowing users to modify node and link colors, apply filters or colors to pathways and tissues when available, and adjust the query ID, edge widths, and node sizes. Furthermore, a new tab has been added to visualize KEGG pathway EA performed using ANUBIX and EASE. These methods were selected because ANUBIX and EASE have demonstrated top speed and performances in our previous benchmark (63).

## Discussion and Conclusions

We present the sixth release of the FunCoup database of functional association networks, where we introduce several methodological advancements and tools designed to enhance network inference and analysis. Key improvements in FunCoup 6 include significantly expanded gene regulatory link coverage, the development of a novel bin-free naïve Bayesian training framework and a comprehensive redesign of the website.

The significant increase in the number of links in FunCoup 6 is driven by multiple factors. First, the inclusion of more and new datasets expanded the underlying data, allowing for greater coverage. Second, the novel bin-free Bayesian training framework improved the granularity and accuracy of link predictions. Additionally, the new link confidence approach provides a key advantage by balancing the support of each gold standard across the network, leading to a more homogeneous and varied representation of functional associations. This combination of factors has not only increased the number of links but also improved the biological relevance of the networks, particularly in species like human, where the number of directed regulatory links has increased substantially.

Despite the use of standardized pipelines and normalized enrichment scores, a limitation of the GRG approach for inferring regulatory links is the inherent complexity in comparing peaks from independent experiments. A peak with a score of 1 in one experiment may not be as biologically significant as a similarly scored peak in another one due to differences in experimental conditions, sequencing depth and other technical factors. However, in a Bayesian integration framework like FunCoup, protein pairs are unlikely to form high-confidence links without support from additional experimental evidence, thereby reducing the risk of directed false positive interactions.

We opted for a bin-free naïve Bayesian training framework for network inference due to its balanced advantages in computational efficiency, interpretability, and its capability to integrate diverse omics data. This bin-free implementation enhances resolution, accuracy, and training stability by avoiding the arbitrary discretization of continuous data, allowing for a more precise representation of the underlying biological signals. While advanced methods such as neural networks excel at capturing complex, non-linear relationships, they require significant computational resources and often lack interpretability. In contrast, the naïve Bayesian network inference method, which assumes conditional independence between data sources, provides an effective means of integrating multi-modal omics data. This makes it an optimal choice for handling the diverse and complex nature of biological datasets encountered in FunCoup 6.

To ensure fair comparison in our benchmark, we applied network propagation on interactomes with a fixed number of links, performing a standardized number of random link swaps to introduce controlled variability. This method preserves the original degree distribution and topological properties of the networks, ensuring that observed performance differences reflect true variations in network structure rather than artifacts from the shuffling process. The use of ORPHANET gene sets, which are not biased towards common diseases, further reinforces the validity of the used benchmark. By using ORPHANET instead of DisGeNET (64) or GWAS data as done in a previous benchmark (19,62) our results came out differently, although in that study FunCoup, HumanNet, and STRING were all top-performing networks with relatively similar performance. However, an important aspect highlighted in Figure 4 is that different networks can provide complementary links. Certain gene sets perform better in one network than another, illustrating the value of combining multiple networks to capture the full complexity of functional associations. This variability in performance underscores the idea that no single network is universally superior, but rather that the diversity of networks adds value by revealing different aspects of gene relationships.

In order to improve user experience and functionality, we developed a new website featuring intuitive advanced search, improved query disambiguation, a D3.js-powered network view with customization options and a new tab for KEGG pathway EA using top-performing methods (ANUBIX and EASE). We also upgraded the API, which now offers functionality that mirrors the capabilities of the website, allowing users to perform searches programmatically and receive results in JSON format. Detailed instructions for using the updated API are available on the API page of the FunCoup website. Additionally, FunCoup is now available as a Cytoscape app (65), which can be accessed and installed at https://apps.cytoscape.org/apps/funcoup.

In addition to the MaxLink tool, the incorporation of the TOPAS algorithm facilitates the identification of disease and drug target modules. We selected TOPAS as part of our in-house ecosystem of tools, which ensures seamless integration, greater control and consistency in our analyses. TOPAS has further been shown to yield superior performance in terms of seed recovery rate, seed-to-connector ratio and module detection consistency (29). Another unique feature of FunCoup is the comparative interactomics tool, which provides insights into network conservation and allows for ortholog alignment or independent species network queries. The bin-free training framework has been applied to 23 primary species, with networks for an additional 618 species in InParanoiDB 9 generated using a novel orthology-based method. Together these enhancements in FunCoup 6 reflect our ongoing commitment to providing a robust and versatile resource for the analysis of functional association networks, facilitating a deeper understanding of complex biological processes and their implications in health and disease.

## Supplementary Material

gkae1021_Supplemental_File

## Data Availability

All data is available at https://funcoup.org, and the code is accessible at https://bitbucket.org/sonnhammergroup/funcoup-6/.
