# Supplementary material for: FunCoup 6: advancing functional association networks across species with directed links and improved user experience
Source: Nucleic Acids Res. 2024 Nov 12;53(D1):D658–71. doi: 10.1093/nar/gkae1021 (PMC11701656; doi:10.1093/nar/gkae1021)
Supplement: gkae1021_Supplemental_File [file gkae1021_supplemental_file.pdf]

Supplementary Materials

SUPPLEMENTARY FIGURES

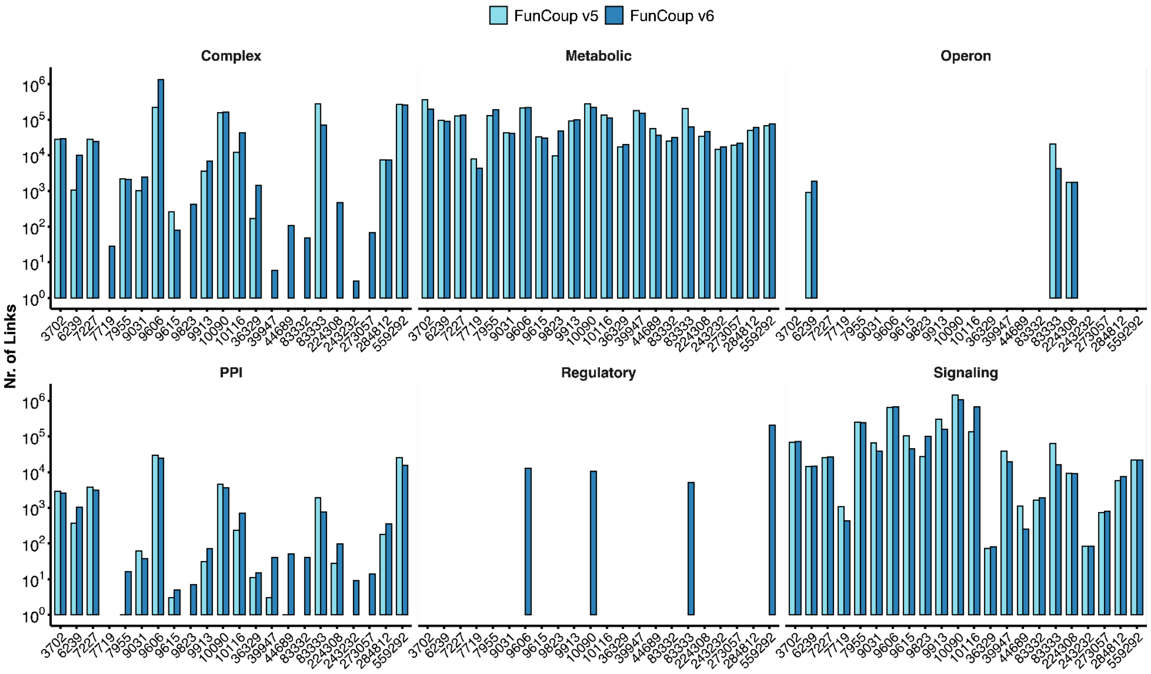

Supplementary Figure 1. Number of links per gold standard across FunCoup species, comparing FunCoup 5 and FunCoup 6. The figure provides a comparison of link counts associated with different gold standards for various species between the two versions.

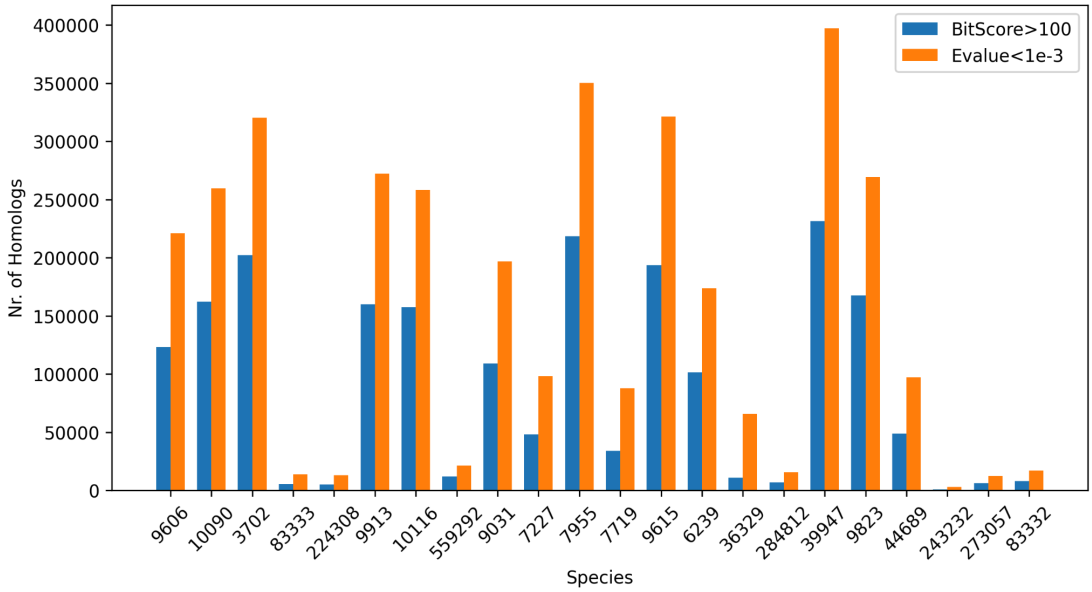

Supplementary Figure 2. Comparison of number of homologs between FunCoup versions. In FunCoup 5 we excluded MEX support for protein pairs that had Blast-based bit scores larger than 100. In FunCoup 6 we consider homologs protein pairs with DIAMOND-based E-value less than 1e-3. Here we compare the number of homologs between DIAMOND-based bit score (*i.e.* which is similar to Blast bit score) and E-value.

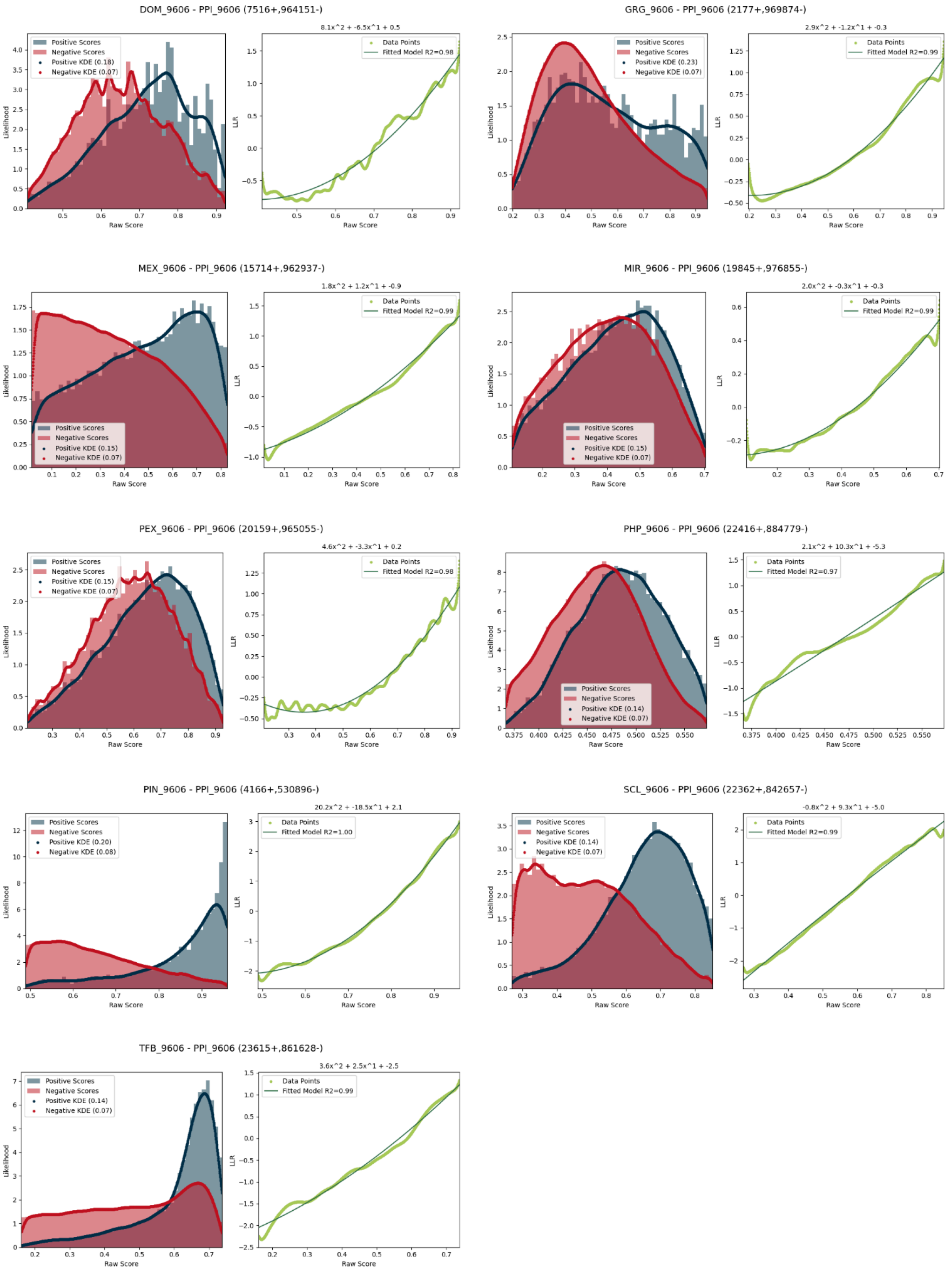

Supplementary Figure 3. Inference of log-likelihoods in bin-free naïve Bayesian network training. Diagnosis plots with Kernel Density Estimation (KDE) of PPI likelihoods (left panel) and polynomial regression of Log-Likelihood Ratios (LLR) (right panel) from 9 out of 10 evidence data in H.sapiens. Training of GIN evidence was unsuccessful with the PPI gold standard due to lack of data (*i.e.* PPI links with GIN score were fewer than 100).

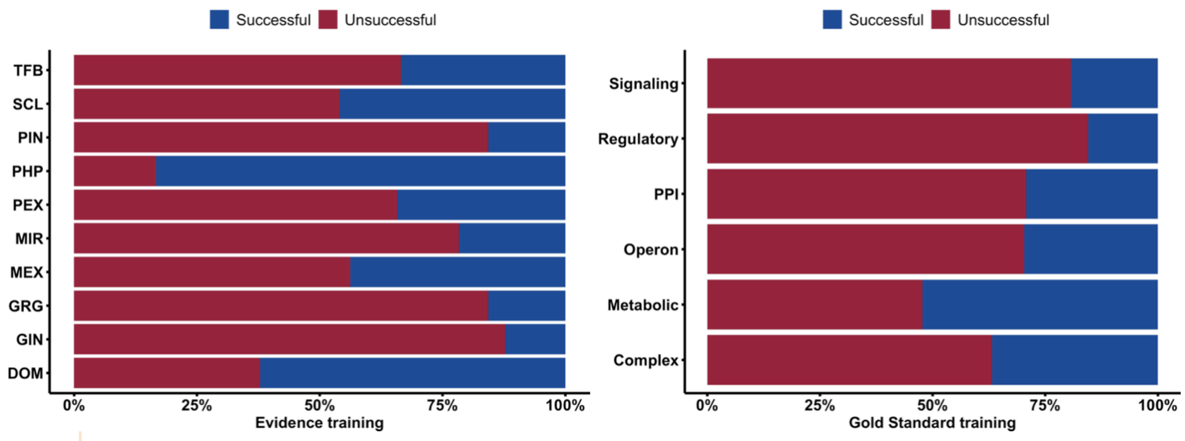

Supplementary Figure 4. Success rate of log-likelihood extraction using bin-free naïve Bayesian network training.

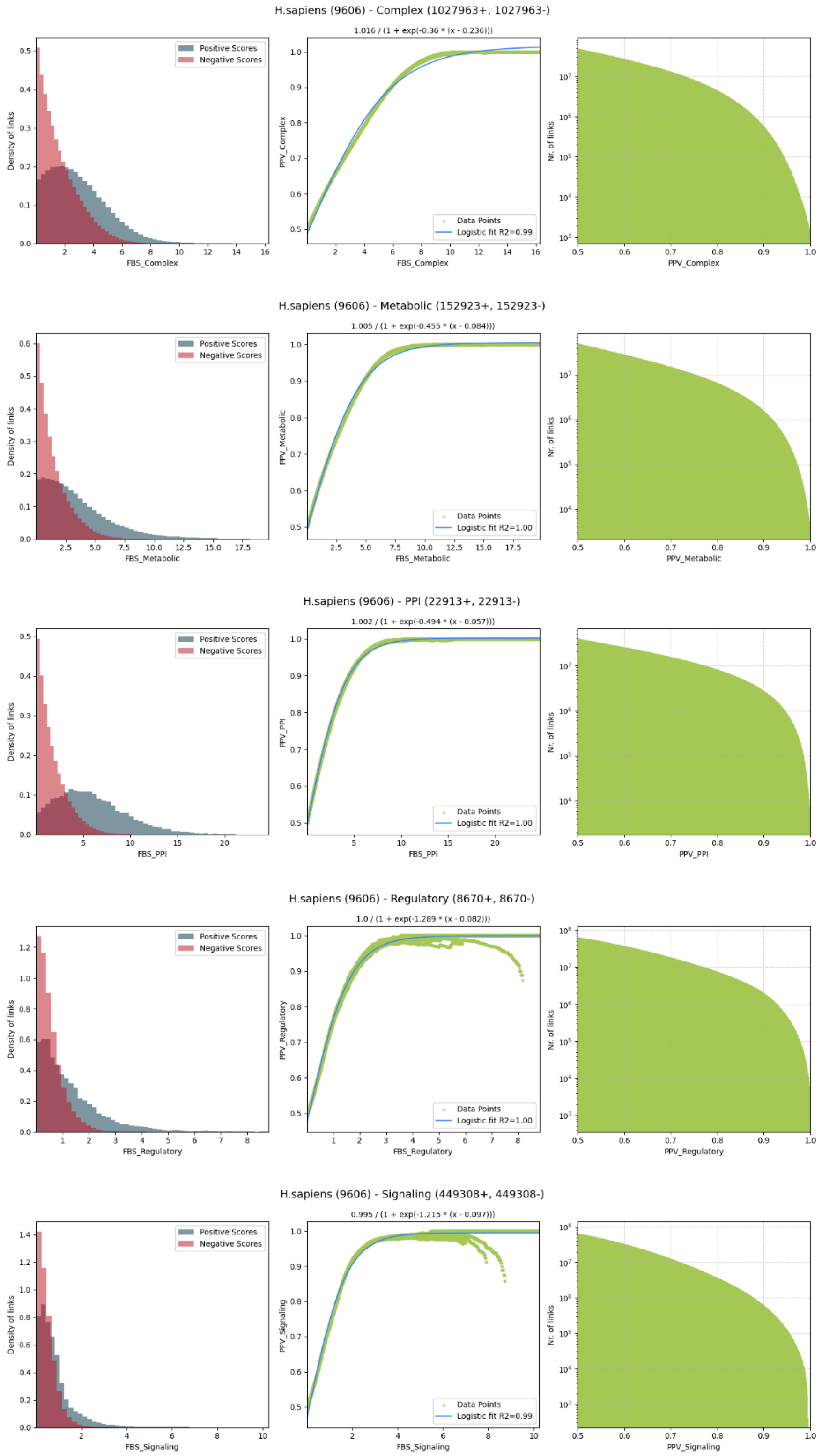

Supplementary Figure 5. Inference of PPV confidence. Diagnosis plots with density of 30x sampled positive and negative examples (left panel), logistic regression of Final Bayesian Score (central panel), and cumulative distribution of number of links (right panel) for all five gold standard networks in H.sapiens.

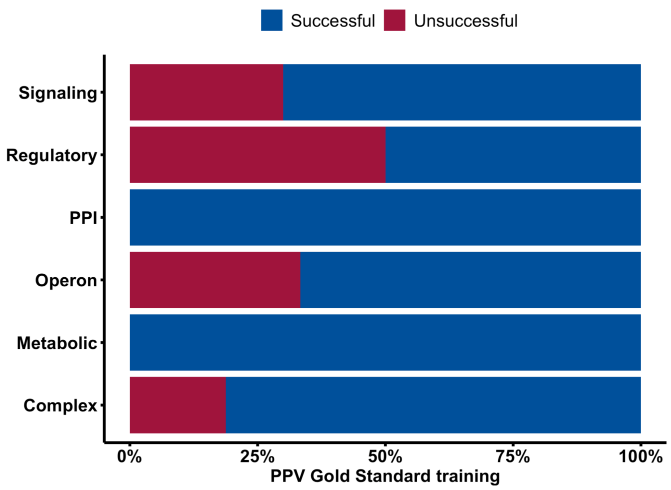

Supplementary Figure 6. Success rate of PPV confidence extraction.

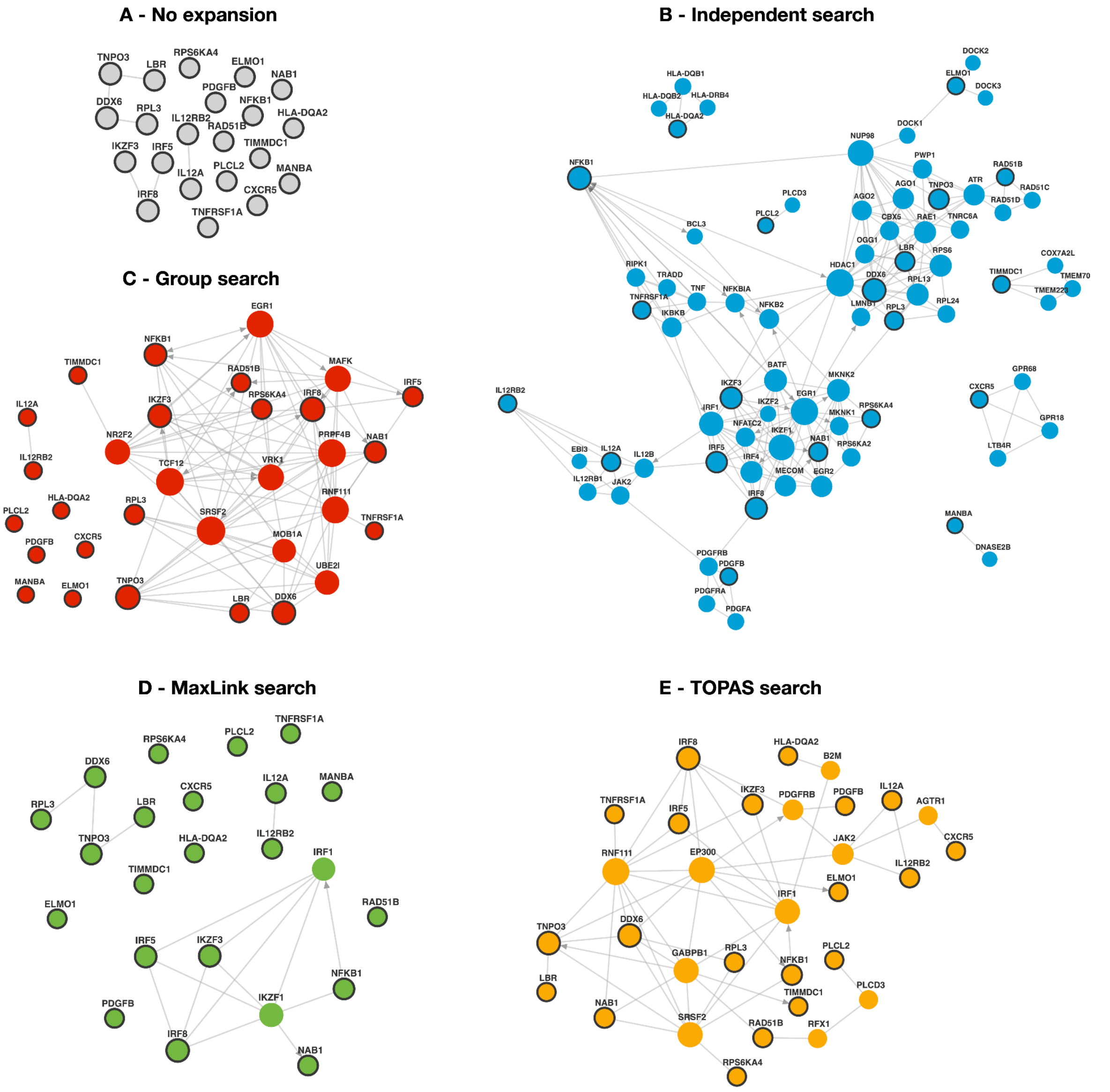

Supplementary Figure 7. Subnetworks of *Biliary liver cirrhosis* using four different search algorithms. The database query (I12R2, NAB1, TIDC1, RA51B, KS6A4, MANBA, PDGFB, DQA2, TNR1A, NFKB1, DDX6, IL12A, CXCR5, RPL3, IRF8, IRF5, LBR, ELMO1, IKZF3, PLCL2, TNPO3) was performed via the website with link and direction confidence thresholds of 0.95 and 1. (A) Only query genes were searched for connections in the network. (B) Genes were queried independently and the top 3 most confident interactions for each individual query gene were added to the network. (C) Genes were searched as a group, with the prioritization of neighbors option activated, and expansion depth of 1 and max 10 neighbors. (D) Gene prioritization via MaxLink, with 15 maximum candidates at a p-value cutoff of 0.05. (E) Disease module detection by TOPAS with 2 maximum allowed connectors in a shortest path between any two query genes. In the network illustration: query genes are highlighted with bold outlines, the size of the nodes is proportional to the degree of connectivity, and nodes are colored using a new website feature.

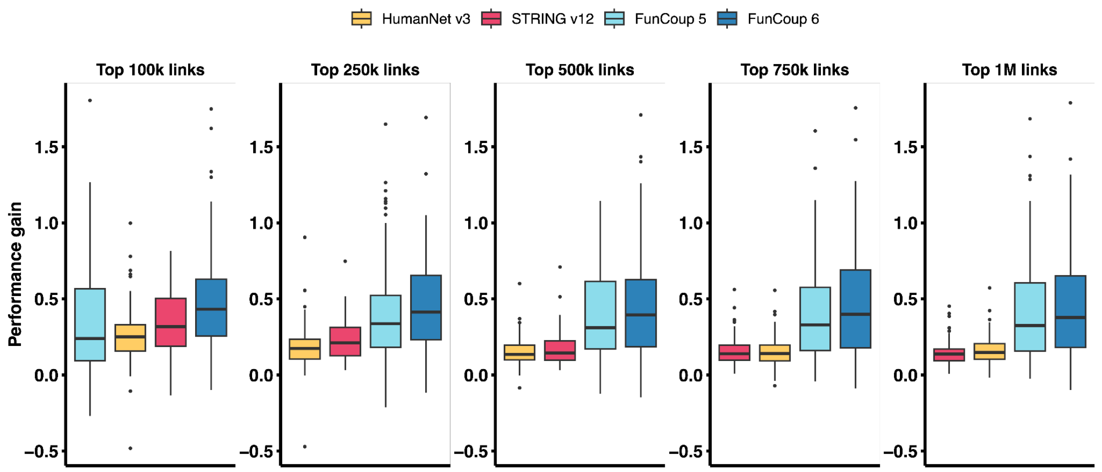

Supplementary Figure 8. Performance gain of networks in the ORPHANET benchmark. Performance gain is quantified as the difference in Area Under the Receiver Operating Characteristic curve (AUROC) between real networks and their null counterparts. The assessment was done using a top fixed number of links: 100,000, 250,000, 500,000, 750,000, and 1.000.000. For top 100,000 links, FunCoup 5 covers 6,934 genes, whereas FunCoup 6 covers 9,734 genes, STRING 12,310 genes, and HumanNet 15,344 genes.

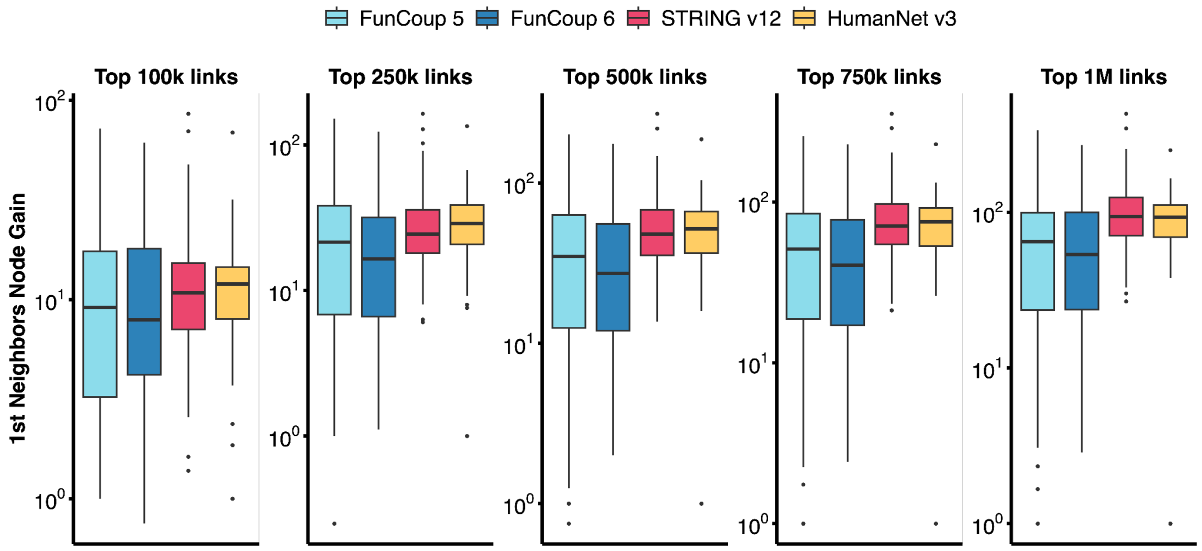

Supplementary Figure 9. First-order neighbor nodes gain distribution across benchmarked networks. The distributions of ORPHANET gene set sizes after mapping them onto the networks using top 100.000, 250.000, 500.000, 750.000 and 1.000.000 most confident links, and 1st-order neighbor node gain, expressed as the ratio of non-query nodes that are 1st-order neighbors to the query genes, and the number of query genes.

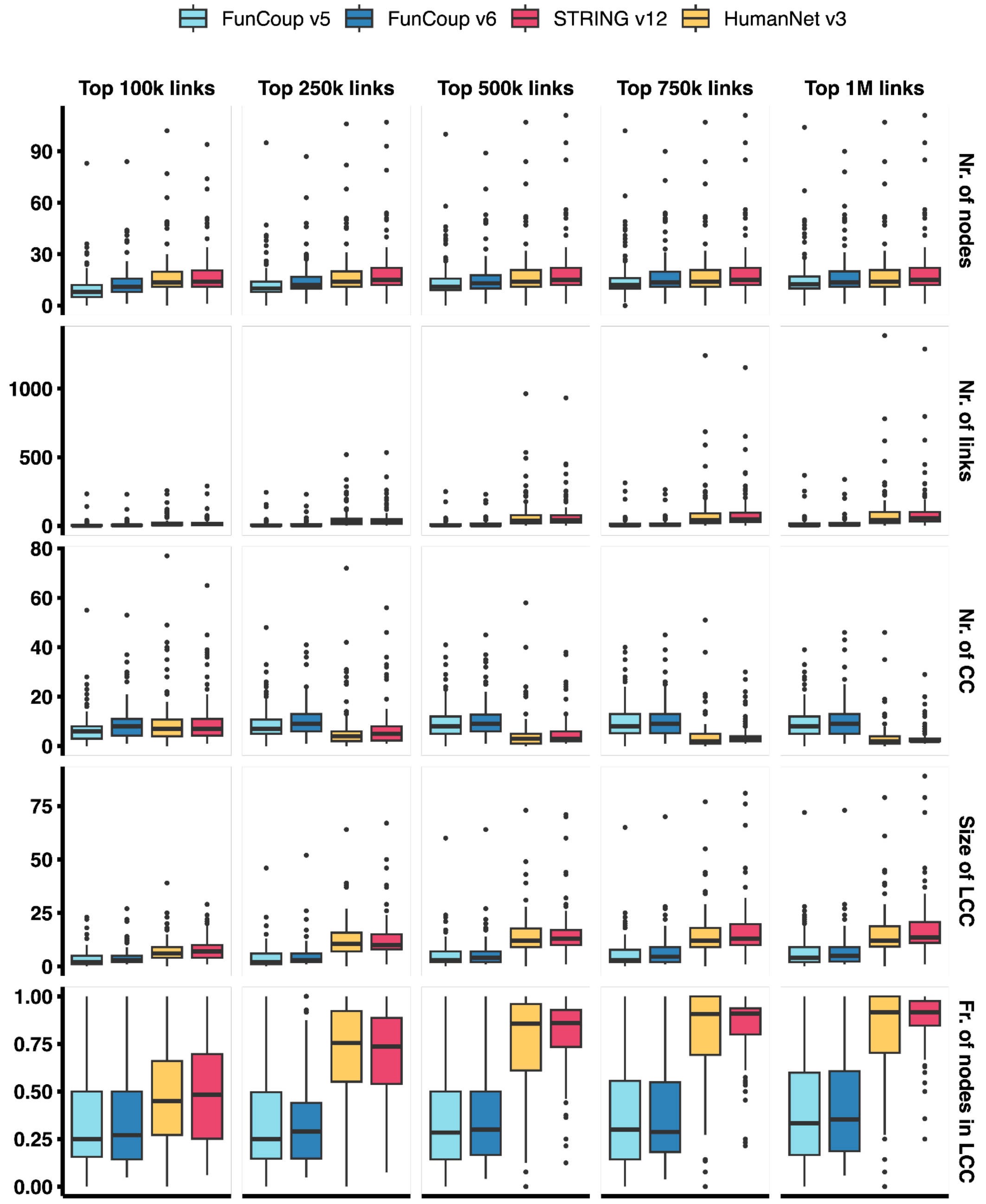

Supplementary Figure 10. Overview of ORPHANET gene set properties across all benchmarked networks. This figure presents key metrics for each network, including the number of nodes, links, connected components (CC), size of the largest connected component (LCC), and the fraction of nodes within the LCC.

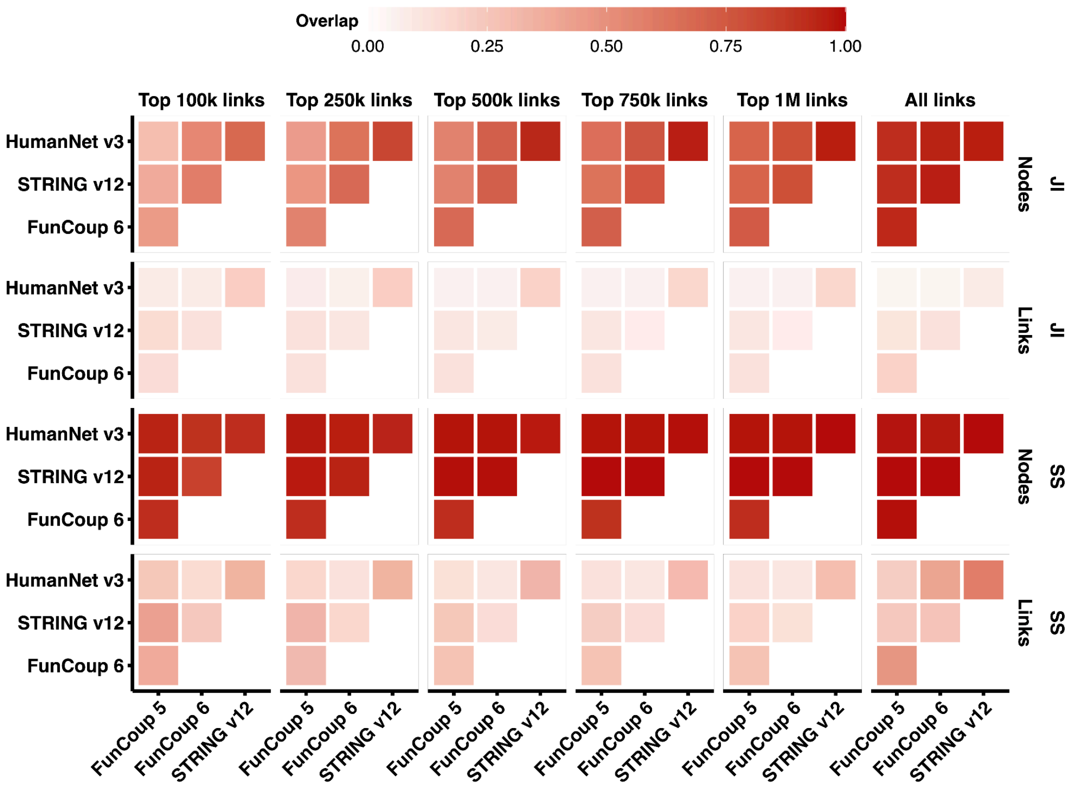

Supplementary Figure 11. Benchmarked network similarity. Similarity between the networks expressed as Jaccard index (JI) and Szymkiewicz–Simpson (SS) coefficient for nodes and links.

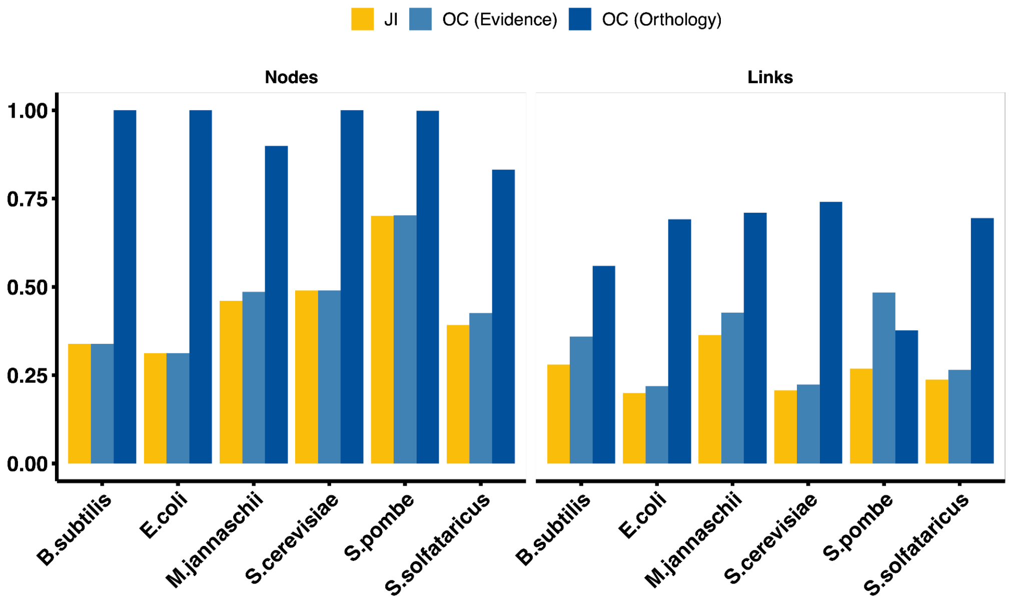

Supplementary Figure 12. Comparison between evidence-based and orthology-transferred networks. To assess the quality of the orthology-transferred networks, we compared them against evidence-based networks for six species using the Jaccard Index (JI) and overlap coefficient (OC) for nodes and links. The overlap coefficient is normalized separately for the evidence-based (Evidence) and orthology-transferred (Orthology) networks, resulting in two distinct values

SUPPLEMENTARY TABLES

Supplementary Table 1. Summary of the novel aspects introduced in FunCoup 6.

Features	FunCoup 6	FunCoup 5
Nr. of species	23 primary + 618 orthology-transferred	22
Nr. of total links	101,054,256	60,999,771
Nr. of directed links	964,037 (13 species)	1,114 (1 species)
Avg. proteome coverage	72%	67%
Primary gene identifier	UniProtKB	Ensembl
SARS-CoV2 / Human network	17 proteins, 2,879 links	23 proteins, 214 links
Training method	Bin-free redundancy weighting naïve Bayesian inference	Binned redundancy weighting naïve Bayesian inference
Disease module detection	MaxLink, TOPAS	MaxLink
Comparative interactomics	Aligning orthologs; independent search	Aligning orthologs
Built-in EA	EA of KEGG pathways with ANUBIX and EASE	-
Website	Python Django	Java FreeMarker
Graph manipulation	D3.js	jSquid
API	RESTful API + Cytoscape app	-
Open source	Yes	-

Supplementary Table 2. Summary of changes to data source and scoring methods for the FunCoup evidences. Differences between FunCoup 5 and 6 for all evidences (i.e. DOM: domain-domain interaction, GIN: genetic interaction; GRG: gene regulation; MEX: mRNA co-expression; MIR: microRNA co-regulation; PEX: protein co-expression; PHP: phylogenetic profile similarity; PIN: physical interaction; SCL: subcellular co-localization; TFB: shared transcription factor binding). Details about the scoring methods can be found in the Methods section.

	Data source		Scoring method	
Evidence	FunCoup 5	FunCoup 6	FunCoup 5	FunCoup 6
DOM	UniDomInt v1.0	UniDomInt v1.0	UniDomInt score	Norm. average weighted UniDomInt score
GIN	Costanzo et al. 2010-2017	BioGRID v4.4.219	pre-computed scores	Spearman correlation
GRG	—	ENCODE v131.0	—	Norm. maximum peak Enrichment Score
MEX	GEO	EBI Expression Atlas, GEO	Absolute Spearman correlation	Absolute Spearman correlation ≥ 0.5
MIR	microRNA.org v2010, MirTarBase	microRNA.org v2010	Jaccard-index like	Jaccard-index > 0
PEX	HPA v19	PaxDb v5.0	Weighted mutual information	Jaccard-index > 0, and Spearman correlation
PHP	InParanoiDB v8.0	InParanoiDB v9.0	Ortholog ratio	Ortholog log ratio
PIN	iRefIndex v16	iRefIndex v2022-08	Average weighted PubMed publications	Norm. average weighted PubMed publications
QMS	PaxDb v4.1	—	Jaccard-index like	—
SCL	GeneOntology v2022-03	GeneOntology v2023-03	Weighted mutual information	Graph-based semantic similarity
TFB	ENCODE, Yeastract 2011-2016	TFLink v1.0	Jaccard-index like	Jaccard-index > 0
